# Multi-locus SNP analyses of interleukin 1 receptor associated kinases 2 gene polymorphisms with the susceptibility to rheumatoid arthritis

**DOI:** 10.1371/journal.pone.0268496

**Published:** 2022-05-19

**Authors:** Maham Ghouri, Muhammad Ismail, Syeda Areesha Zaidi, Shafique Rehman, Asadullah Dahani, Saima Saleem, Sitwat Zehra

**Affiliations:** 1 The Karachi Institute of Biotechnology and Genetic Engineering (KIBGE), University of Karachi, Karachi, Pakistan; 2 Institute of Biomedical and genetic Engineering (IBGE), Islamabad, Pakistan; 3 Jinnah Post Graduate Medical Center Karachi (JPMC), Karachi, Pakistan; Unicamillus, Saint Camillus International University of Health Sciences, ITALY

## Abstract

**Background:**

The genetic polymorphisms (*rs708035*, *rs3844283)* of Interleukin-1 receptor associated kinases 2 (IRAK2) is involved in the NFκB regulatory pathway. The frequencies of *IRAK2* gene are unknown in Pakistani population. Therefore, the study was designed to examine the association of targeted single nucleotide polymorphism(s) in *IRAK2* gene of RA patients.

**Methodology:**

The study participants were selected by ACR/EULAR 2010 standards. After ethical approval, the blood samples of patients and healthy controls were collected for the extraction of DNA followed by the amplification of targeted polymorphism(s) via Tetra-primer Amplification Refractory Mutation System (T-ARMS PCR). Desired products were observed via agarose gel electrophoresis.

**Results:**

The allele frequency of wild type A and C is frequent among patients and mutant T and G is frequent among controls. The *rs708035* showed significant protective association while *rs3844283* was found to be associated with risk of RA. Genetic model associations were applied to determine the role of genotypes. In combination analyses of alleles revealed AC haplotype was found to be associated with risk and TG provide protection against RA. Moreover, targeted SNPs were found to be in 61% Linkage Disequilibrium among the targeted population.

**Conclusions:**

Current study revealed the protective and risk association of targeted SNPs (*rs708035*, *rs3844283)*. Study might be beneficial as it provides baseline data regarding targeted SNPs and their role in the disease progression. This could be served as potential biomarker for diagnostic purpose and effectively utilized in precision medicine approach.

## Introduction

The IRAK2 (Interleukin-1 receptor associated kinases 2) interacts with T cells in the presence of IL-1 (Interleukin-1) which releases signals to initiate immune response against foreign particles. RA (Rheumatoid Arthritis) is an autoimmune disease with unknown etiology. There are various risk factors which may be responsible for the disease progression like obesity, previous infections, habit of smoking and history of joint injuries [1]. According to reports by WHO (World Health Organization) RA affects 1% of the population globally [2]. Developing countries like Pakistan do not have much data due to lack of repository related to RA, but various tertiary hospitals in Pakistan proposed that the prevalence in the southern region and northern regions was 0.142%—5.5% respectively [3]^.^ There are various proinflammatory cytokines involved in the pathophysiology of RA which are IL-23, IL-1 and TNFα.

The NFκB (The nuclear factor kappa light chain enhancer of the activated B cell mediated activation of cytokine gene expression) works as transcriptional factor for the release of these targeted cytokines [4–6]. NFκB works in the form of cascade initiates by various IRAKs (Interleukin receptor associated kinases). The Phosphorylated form of IRAKs were separated from the receptor complex and binds to the TRAF6 [TNF receptor associated factor (TRAF) protein family] within the cytoplasm [7, 8]. The TRAF6 also stimulates TAK-1 (transforming-growth-factor-β‐activated-kinase *1*). The successive stimulation of IKK (IκB kinase) complex downregulate NF-κB. Once the level of IκBα is decrease it translocate NF-κB dimers to the nucleus from the cytoplasm to increase the level of NF-κB. The pathway progresses in the form of cascade of reactions which is accomplished by the polyubiquitination of *IRAK2* [9, 10].

*IRAK2* encodes for the IRAK2 enzyme which is the one of the putative serine/threonine kinases responsible for the regulation of NFκB [11, 12]. As a result of this immunity oxidative stress is generated within the body in the form of ROS (reactive oxygen species) which also contributes to the development of disease by inducing apoptosis, mitochondrial dysfunction, increase synovial inflammation, cartilage degradation, chondrocytes senescence [13, 14].

Several studies revealed the availability of limited treatment options against RA to minimize symptoms by suppressing person’s immunity. Various side effects of currently available treatments are reported by the FDA (Food and Drug Administration) including increased risk of high blood pressure, heart failure and liver problems which may lead toward life threatening allergic reactions. The SNPs (*rs708035* and *rs3844283*) are responsible in increase and decrease of NFκB levels either by promoting or reducing polyubiquitination of TRAF6 [15]. Based on prior studies current study was planned to find out the association of SNPs (*rs708035* and *rs3844283)* with *IRAK2* gene that might be responsible for progression of RA. It would also be beneficial in understanding the development and progression of the disease by providing base line data for the Pakistani population.

## Methodology

### Selection of study participants

Studied participants were selected following ACR/EULAR 2010 standards mentioned in S1 Table [1]. The study was conducted on 1000 participants, 500 diagnosed RA patients and 500 healthy controls. Ethical approvals from institutional ethics committee of The Karachi Institute of Biotechnology and Genetic Engineering (KIBGE), University of Karachi [Ref No. KIBGE/ICE/301/10/09/2018] and Jinnah Postgraduate Medical Center (JPMC) [NO.F.2-81-IRB/2019-GENL/19855/JPMC] was taken from their respective IRB (Institutional Review Board). Patients with confirmed diagnosis of RA were included in this study. Patients with other type of arthritis, pregnant women, diabetes, and other autoimmune disorders were excluded from the study. Selection of controls were made prior to one-month with no symptoms of inflammation and individuals with history of any inflammatory disease was excluded. After taking written inform consent blood samples from participants were collected. The Patient’s general history was recorded including their diagnostic tests and patient’s characteristics.

### DNA isolation & SNP selection

Based on minor allelic frequency (MAF), Single Nucleotide Polymorphisms (*rs708035* and *rs3844283*) were selected. Selection was done using Ensembl genome browser 92 where the MAF of targeted SNPs were greater than 0.05 among the pooled populations [16]. Ensembl genome browser 92 covers the population from (African American, European, and Sub-Saharan African) [17] and the NCBI SNP data base [12]. The gDNA was extracted by optimized protocol of salting out method.

### Amplification of targeted polymorphism(s)

Amplification of targeted polymorphism(s) *rs708035* and *rs3844283* were achieved by optimized protocol of T-ARMS PCR, two set of primers were designed by Primer1 software [18]. The sequences of primers are mentioned in S2 Table. PCR reaction cycle was performed on Bio-Rad PCR Machine for both SNPs. Targeted regions of *IRAK2* were than amplified by initial denaturation at 95°C for 5 minutes followed by separate denaturation at 95°C for 30 seconds. Primers were annealed at 60.3°C (***rs708035)*** and 63.7°C (***rs3844283***) for 45 seconds following the extension at 72°C for 45 seconds to elongate targeted region. After each step additionally RAMP temperature was provided at 2.5°C/Sec. Cycle was repeated for 35 times and stopped at final elongation temperature at 72°C for 8 minutes and stored at 4°C. Desired product was visualized on gel electrophoresis using 3% gel (Fig 2).

### Bioinformatics & statistical analyses

Association analyses was performed by applying chi-square test using SPSS version 23 (SPSS Inc., Chicago, IL, USA). Risk ratio of SNPs was calculated by determining odds ratios (OR) and 95% confidence intervals using online software MedCalc [19]. The SNP-stats software was used to determine genotype associations SNP via genetic models. On the other hand, the genotype models were applied to check the strength of association by using online available software SNPStats [20] where p < 0.05 was considered as statistically significant. The Linkage Disequilibrium was performed by using online available software SHEsis [21].

## Results

### Patients’ characteristics

**Fig 1:**
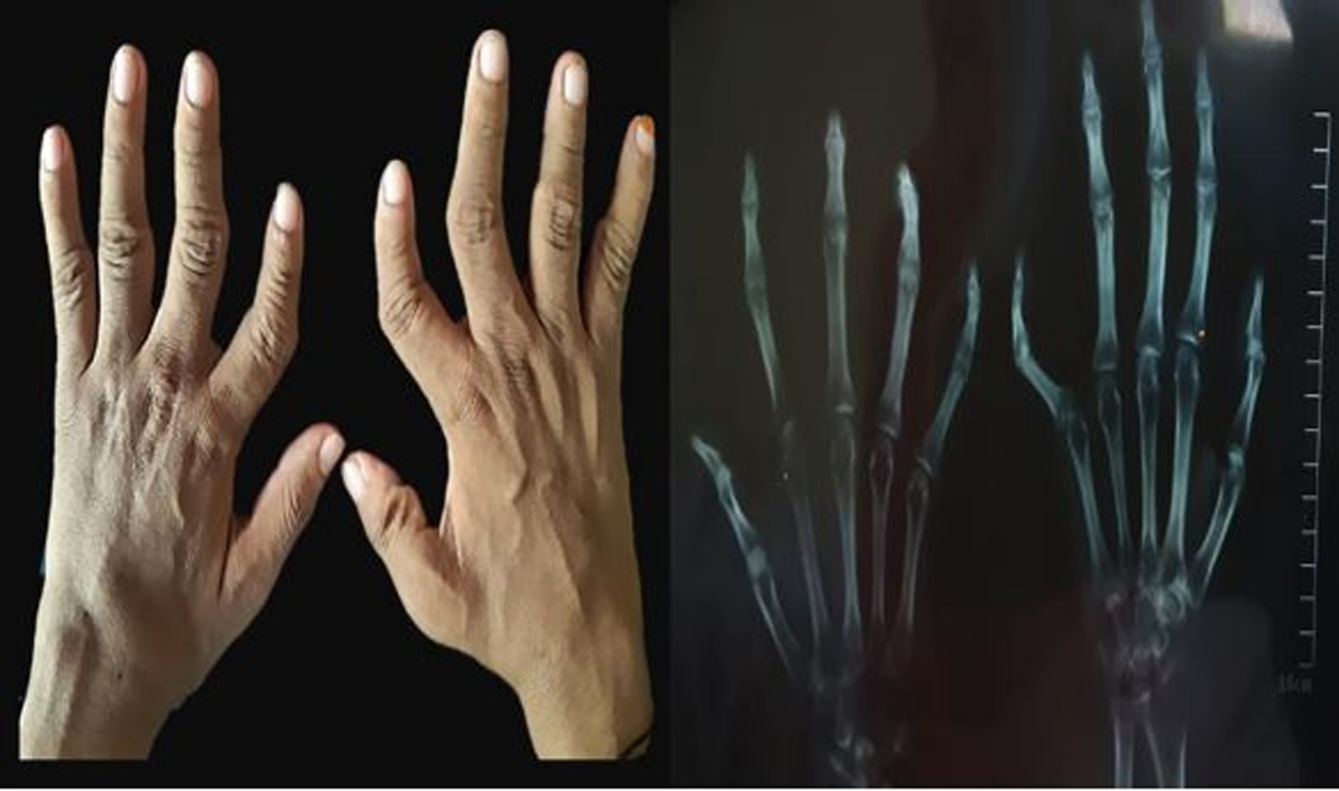
Physical feature of hand. Physical appearance of patient’s hands depicted that patient suffers from severe joint pain.

Patients suffering from rheumatoid arthritis were facing severe health issues which mostly effects their hand’s joints (Fig 1). Among patients’ females were found be more affected than males (85% and 15% respectively). The patient’s characteristics including age, Body mass index (BMI), Erythrocyte Sedimentation Rate (ESR), C-reactive protein (CRP), RA Factor, anti-citrullinated protein antibodies (ACPA), Tenderness in Joints Count (TJC), Swelling in Joints Count (SJC), General Health Form (GHF), DAS28ESR, DAS28CRP and Disease duration of disease were significant among the group of patients. (Table 1).

**Table 1:** Patients’ demographic data.

Characteristic	Mean ± SEM Controls (n = 500)	Reference Values	Mean ± SEM Patients (n = 500)
**Age (years)**	40.22±0.49	N/A	41.19±0.513***
**BMI (kgm** ^**-2**^ **)**	24.10±0.216	N/A	24.09±0.236***
**ESR (mmhr** ^**-1**^ **)**	N/A	Male <15 Female <20	51.02±1.082***
**CRP (mgL** ^**-1**^ **)**	N/A	<5	19.67±1.084***
**RA Factor (IUmL** ^**-1**^ **)**	N/A	<14	55.73±3.397***
**ACPA (UmL** ^**-1**^ **)**	N/A	<20	323.47±37.348***
**Tenderness in Joints Count**	N/A	>8 Joints out of 28	15.66±0.257***
**Swelling in Joints Count**	N/A	>8 Joints out of 28	24.60±0.232***
**General Health Form**	N/A	>50	60.97±0.782***
**DAS28CRP**	N/A	>5	6.26±0.036***
**DAS28ESR**	N/A	>5	7.03±0.037***
**Duration of Disease (Months)**	N/A	N/A	90.16±4.275***

Patient’s history related to disease progression showing in Mean ± Standard error of mean (SEM). One sample statistic showing the significance score

(***) p<0.001, values without steric are non-significant and (N/A) not applicable.

### Single SNP analyses (*rs708035* and *rs3844283)*

**Fig 2:**
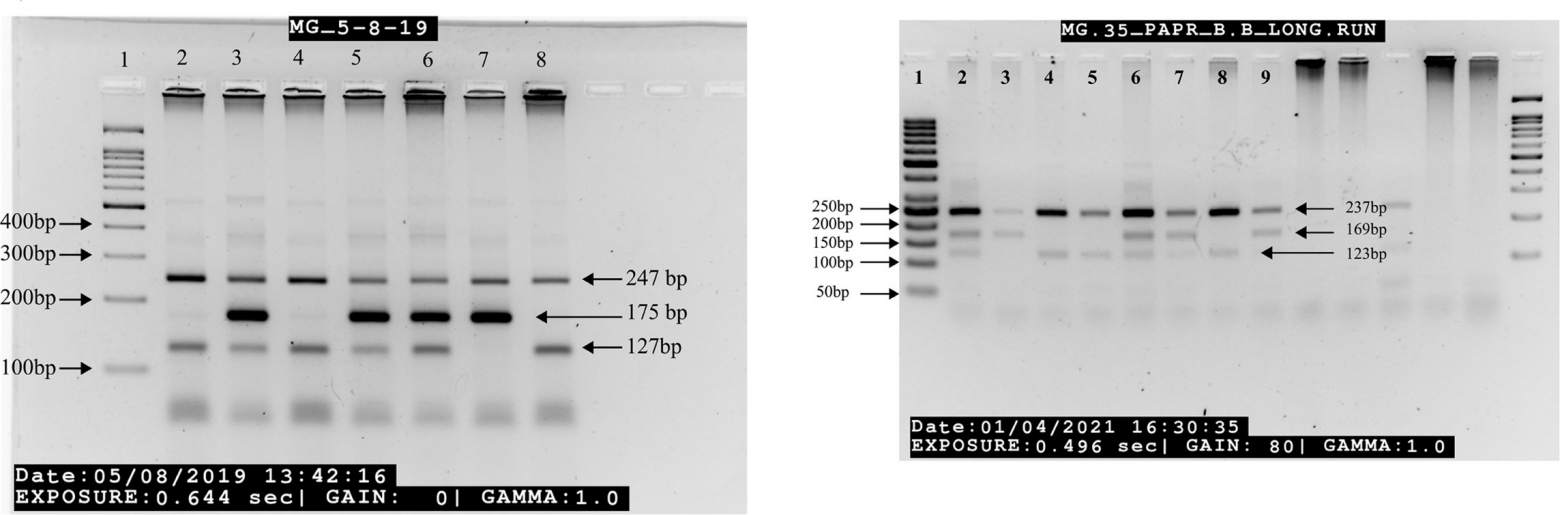
Amplified product on agarose gel (3%). A. lane 2 to 8 showed genotypes of SNP *rs3844283* where two bands indicating homozygous genotype and three bands indicates heterozygous genotypes. Lane 1 carrying 100bp marker, Lane 2 and 8 indicating C/C wildtype genotype, Lane 3 to 6 having heterozygous C/G genotype with three bands and Lane 7 showing G/G mutant genotype. B. Lane 1 to 9 from left showed genotypes of SNP *rs708035* where two bands indicating homozygous genotype Lane-1 carrying 50bp marker, lane 2, 6 and 7 indicating Heterozygous A/T genotype with three bands, lane 3 and 9 showing homozygous mutant genotype T/T, lane 4,5 and 8 carrying homozygous wildtype genotype A/A.

The amplification of desired region was observed on the 3% agarose gel (Fig 2A and 2B). The allelic frequency of *rs708035* wild type A allele was frequent among cases while the frequency mutant T allele was frequent among controls. The wild type C allele of *rs3844283* was frequent in patients while mutant G allele was frequent in controls. The SNP *rs708035* was significantly associated with the protective role against RA [χ^2^ = 28.676, p = <0.01, O.R 95% CI = 0.517, (0.405~0.66), p = <0.01]. The SNP *rs3844283* was significantly associated with the risk of RA [χ^2^ = 4.273, p = <0.05, O.R 95% CI = 1.204 [1.009~1.436], p = <0.05] (Table 2). The genotype A/A was frequent among patients while genotype T/T and heterozygous A/T genotype was frequent in controls. On the other hand, the genotype C/C and G/G is frequent among controls while the heterozygous C/G was found to be frequent in controls (Table 2).

**Table 2:** Association analyses of SNPs *rs708035* and *rs3844283*.

***rs708035***						
**Genotypes**	**Patients (n = 500/1000)**		**Controls (n = 500/1000)**		**Chi-Square (χ** ^**2**^ **)**	**O.R (95%CI)**
Genotypes Frequency	A/A	384 (77%)	A/A	301 (60%)	32.136***	0.517*** [0.405~0.66]
	A/T	111 (22%)	A/T	188 (38%)		
	T/T	5 (1%)	T/T	11 (2%)		
Allelic Frequency	A	879 (88%)	A	790 (79%)	28.676***	
	T	121 (12%)	T	210 (21%)		
***rs3844283***						
**Genotypes**	**Patients (n = 500/1000)**		**Controls (n = 500/1000)**		**Chi-Square (χ** ^**2**^ **)**	**O.R (95%CI)**
**Genotypes Frequency**	GG	71 (14%)	GG	232 (46%)	304.889***	1.204* [1.009~1.436]
	GC	384 (77%)	GC	108 (22%)		
	CC	45 (9%)	CC	160 (32%)		
**Allelic Frequency**	G	526 (53%)	G	572 (57%)	4.273*	
	C	474 (47%)	C	428 (43%)		

The single locus association test of SNPs (*rs708035*, *rs3844284*) showing genotype and allelic Chi-Square, O.R, 95% CI, P-Value, where

(***) is showing p< 0.001 and

(*) indicating p<0.05.

The strength of association was analyzed by genetic models also confirmed the associations of targeted genotypes (*rs3844283* and *rs708035*) with the risk of disease. The heterozygous A/T genotype was significantly associated with the risk of RA in all genetic models Codominant, Dominant and Over dominant models with [O.R 95% CI = 2.11, [1.59~2.79], p = <0.0001], [O.R 95% CI = 2.09, [1.58~2.76], p = <0.0001] and [O.R 95% CI = 2.08, [1.57~2.76], p = <0.0001] respectively. The multiplicative model (log of additives) also confirms the significant role of all model types [O.R 95% CI = 1.95, [1.50~2.54], p = <0.0001]. On the other hand, the genotype G/G-C/G + C/C confirms the significant association of SNP with risk of RA in recessive model [O.R 95% CI = 5.28, [3.66~7.60], p = <0.0001] (Table 3).

**Table 3:** Genetic model-based analyses of SNPs *rs3844283* and *rs708035* among groups.

**SNP *rs3844283 (n = 1000)***						
**Models**	**Genotype**	**Controls**	**Cases**	**OR (95% C.I)**	**AIC**	**BIC**
**Codominance Model**	G/G	232 (46.4%)	71 (14.2%)	1.00	1035.7	1055.3
	C/G	108 (21.6%)	384 (76.8%)	0.08 (0.06–0.11) ***		
	C/C	160 (32%)	45 (9%)	1.19 (0.77–1.83)		
**Dominant Model**	G/G	232 (46.4%)	71 (14.2%)	1.00	1244.9	1259.6
	C/G-C/C	268 (53.6%)	429 (85.8%)	0.19 (0.14–0.26) ***		
**Recessive Model**	G/G-C/G	340 (68%)	455 (91%)	1.00	1275.5	1290.2
	C/C	160 (32%)	45 (9%)	5.28 (3.66–7.60) ***		
**Over Dominant**	G/G-C/C	392 (78.4%)	116 (23.2%)	1.00	1034.3	1049
	C/G	108 (21.6%)	384 (76.8%)	0.07 (0.05–0.10) ***		
**Log-additive**	--------	--------	--------	0.86 (0.72–1.02)	1366.7	1381.5
SNP *rs708035 (n = 1000)*						
Models	**Genotype**	**Controls**	**Cases**	**OR (95% C.I)**	**AIC**	**BIC**
**Codominance Model**	A/A	301 (60.2%)	384 (76.8%)	1.00	1343.8	1363.5
	A/T	188 (37.6%)	111 (22.2%)	2.11 (1.59–2.79) ***		
	T/T	11 (2.2%)	5 (1%)	1.75 (0.58–5.25)		
**Dominant Model**	A/A	301 (60.2%)	384 (76.8%)	1.00	1341.9	1356.6
	A/T-T/T	199 (39.8%)	116 (23.2%)	2.09 (1.58–2.76) ***		
**Recessive Model**	A/A-A/T	489 (97.8%)	495 (99%)	1.00	1369.4	1384.1
	T/T	11 (2.2%)	5 (1%)	1.37 (0.48–4.08)		
**Over Dominant**	A/A-T/T	312 (62.4%)	389 (77.8%)	1.00	1342.9	1357.6
	A/T	188 (37.6%)	111 (22.2%)	2.08 (1.57–2.76) ***		
**Log-additive**	--------	--------	--------	1.95 (1.50–2.54) ***	1344	1358.7

Genetic models reveled the strength of association where OR < 1 = Protective role of allele, OR = 1 No relationship, OR>1 = Risk allele. The selection of fitted model is based on AIC (Akaike information criterion) and BIC (Bayesian information criterion) and

(***) representing p<0.001.

### Multi-SNP Analyses of SNPs *rs3844283* and *rs708035*

**Fig 3:**
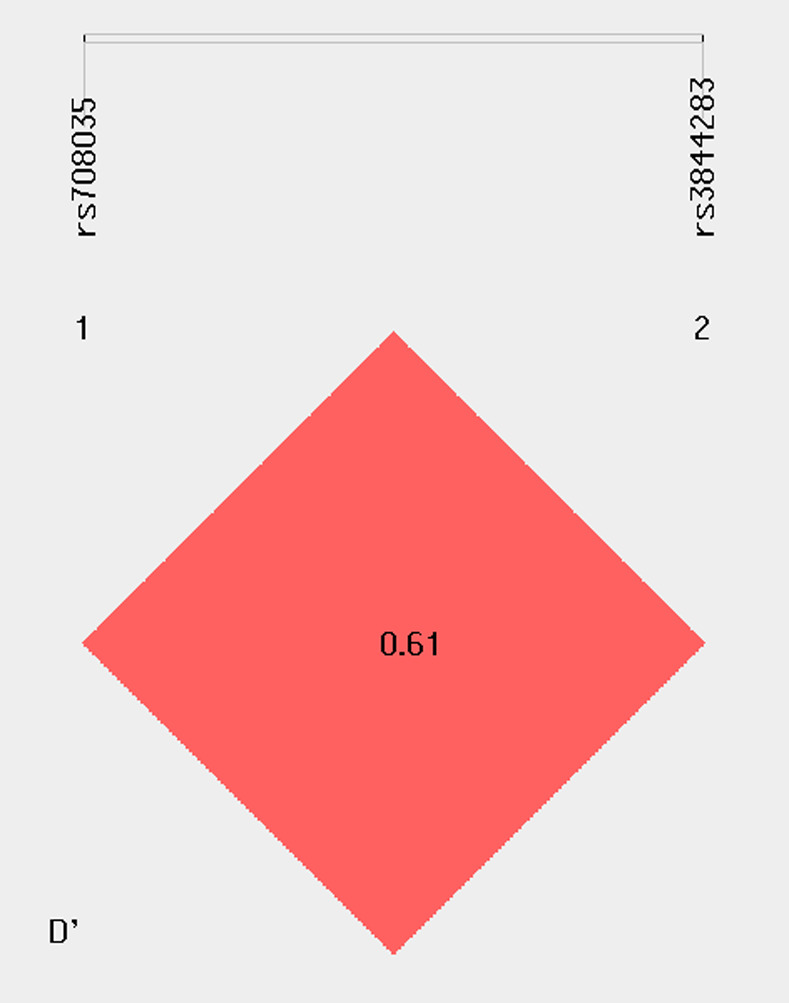
Linkage disequilibrium (LD) analyses for SNP *rs3844283* and *rs708035*. The LD Plot standard plot which showed 61% linkage disequilibrium among targeted SNPs with R^2^ = 0.06.

The combination of alleles at multiple loci was studied to analyze the association with RA. It was observed that the haplotypic frequency of AC was higher in cases and significantly involved in the risk of disease [O.R 95% CI = 1.25, [1.08~1.547], p = <0.01]. On the other hand, the frequency of TG haplotype was frequent among controls which was significantly associated with the protective role against RA [O.R 95% CI = 0.53, [0.412~0.698], p = <0.001] (Table 4). The established Linkage disequilibrium (LD) plot revealed showing targeted SNPs were in 61% LD with D’ = 0.61 (Fig 3).

**Table 4:** Haplotype analysis (chosen loci *rs708035* and *rs3844283*).

Haplotype	Control (Frequency)	Case (Frequency)	Chi-Square (χ^2^)	O.R [95%CI]	Fisher’s p-value	Pearson’s p-value
**AC**	391 (0.391)	454 (0.454)	8.133	1.295^*^ [1.084~1.547]	**0.004**	**0.004**
**AG**	399 (0.399)	425 (0.425)	1.395	1.113 [0.931~1.33]	0.256	0.237
**TG**	173 (0.173)	101 (0.101)	21.923	0.537*** [0.412~0.698]	**3.50e-06**	**2.84e-06**

The multi-locus association test of SNPs (*rs708035*, *rs3844284*) showing haplotype frequencies, Chi-Square and O.R, 95% CI, P-Value, where (****) is showing p< 0.001 and

(***) indicating p<0.01.

## Discussion

RA is the disease that affects small joints which occur due to hyperactivity of immune system. Immune cell arises within synovium, results in joint-impairment and bone-destruction. Once it progressed to the chronic stage this leads to severe disability and enhance-mortality rate among the patients [22]. Such patients deal various consequences, and their quality of life is reduced due to constant pain in joints, weaken joints and eventually death [23]. Since current diagnostic procedures and treatment are limited therefore, it required more genetic research that could help in the early diagnosis of disease or utilize in precision-medicine based treatments. The current study also confirms the progression of disease which results in various physical changes in joints (Fig 1) [24]. Recommendations proposed by the EULAR plays vital role in regular clinical analyses of damage in patient’s joint and inflammation. Hence, patients were selected by using EULAR criteria mentioned in S1 Table [1, 24, 25]. Studied participants suffering from RA were faced similar health issues like hands and feet joint damages with change in physical features. Various studies suggested that the rate of onset of disease is three times higher in females as compared to males, it might be due to various hormonal changes and menopause in females [26]. The significance of observed patient’s characteristics was calculated as MEAN±SEM (Table 1) and results were also supported by other studies [27–29].

*IRAK* is effectively involved in the regulation of TLR/IL1 (interleukin 1 arbitrated toll like receptors) which provide innate immunity to the body. It is reported that *IRAK2* polymorphism(s) *rs708035* and *rs3844283* were found to be involved in the progression of RA among the Egyptian population. These SNPs are responsible to mediate NF-κB transcriptional factor levels to release of pro-inflammatory cytokines which keep patient’s immune system hyper activated [30]. Current findings satisfied the results of previous study regarding *rs708035* allelic frequency [30]. The product size was observed on 3% agarose gel by electrophoresis (Fig 2A and 2B).

In association analyses of targeted SNPs, it was observed that among both targeted SNPs *rs708035* was found to have protective role while *rs3844283* was involved in the risk of RA (Table 2). The SNP *rs708035* does not follow Hardy-Weinberg Equilibrium (HWE) while *rs3844283* was found to be in HWE [30]. It was observed that genotype A/T was significantly involved with the risk of RA in codominant and over-dominant models (Table 3). Among all models’ over-dominant model was selected as statistical fitted model due to quantifiable genetic action where the heterozygosity was frequent over homozygote and has lowest AIC and BIC difference. The genetic model results suggested that one of the alleles carry strong protective role against RA. The genetic model revealed that A allele might be responsible for providing protective role and allele T is responsible for risk association i.e., odds ratio > 1 in all models which is masked by the wild type A allele. In previous study genetic model was not applied to the SNP *rs708035* which was reported as first study published the association of *IRAK2* polymorphisms to RA [30]. The SNP *rs3844283* was significantly associated with the risk of RA. The genetic model association for SNP *rs3844283* revealed that C/C genotype significantly associated with the risk of RA in recessive model with the [O.R 95% CI = 5.28 (3.66–7.60, p<0.001)] while the allele G is responsible for providing protective role in dominant and over dominant models therefore C allele is responsible for risk association. Based on statistical fittest model recessive model was selected due to least difference between AIC and BIC values (Table 3). The results were consistent for the *rs3844283* while odds ratio was not provided for *rs708035* [30]. Since the biological role of targeted SNPs are known, the rs708035 is providing protection as it is involved enhancing the NF-κB transcriptional factor. These targeted SNPs might play crucial role in the diagnosis of disease act as biomarker for the early diagnosis of disease and information might be helpful in precision medicine.

The haplotype frequencies also confirm the protective role of GT haplotype which was frequent among controls while AT was responsible for the disease progression among RA patients (Table 4). The multi SNPS analyses suggested that targeted SNPs were in 61% linkage disequilibrium with D^’^ = 0.61 (Fig 3). The results of multi-locus association test of SNPs were not consistent with the previous study due to the differences in ethnicity [30]. This confirms individual association of *rs708035* in protection and *rs3844283* with the risk of disease. However, the association might be due to linkage disequilibrium, in future further studies with larger sample size in extension with the current study will be required for the confirmation of results.

Since various studies confirmed the linkage of different SNPs with RA progression [31]. The role of targeted polymorphism(s) mentioned in (S1 Appendix). This study provides baseline data regarding targeted SNPs and their role in the disease which could serve as biomarker for diagnostic purpose in future. More studies need to be done at haplotype level by considering neighboring SNPs within the *IRAK2 gene* to explore role of gene in the development of RA. Furthermore, the functional analyses of targeted polymorphism(s) could be explored by introducing D431E and L392V mutation in *IRAK2* of animal models via site directed mutagenesis. Current study might be helpful to determine any change in immune pathways specifically related to transcriptional factor NF-κB since their levels are upregulated among the RA patients at chronic stage [15].

## Supporting information

S1 Table ACR/EULAR criteria 2010 for the selection of patients. (PDF)

S2 Table Primer sequences for SNPs (*rs3844283* and *rs708035)* amplification. (PDF)

S1 Appendix Role of SNP *rs3844283* and *rs708035*. (TIF)
