# Supplementary material for: Multi-locus SNP analyses of interleukin 1 receptor associated kinases 2 gene polymorphisms with the susceptibility to rheumatoid arthritis
Source: PLoS One. 2022 May 19;17(5):e0268496. doi: 10.1371/journal.pone.0268496 (PMC9119434; doi:10.1371/journal.pone.0268496)
Supplement: S1 Table — (PDF) [file pone.0268496.s001.pdf]

S1 Table. ACR/EULAR Criteria 2010 for selection of patients		
**A**	Joint involvement	0-5
**B**	Serology either RF or ACPA (at least one test result is needed for classification)	0-3
**C**	Acute phase reactants either CRP or ESR (at least one test result is needed for classification)	0-1
**D**	Duration of symptoms	0-1

Indicating the selection criteria for patients using ACR/EULAR Criteria 2010, in which patient score ≥ 3 is consider as RA patient.
