# Supplementary material for: Multi-locus SNP analyses of interleukin 1 receptor associated kinases 2 gene polymorphisms with the susceptibility to rheumatoid arthritis
Source: PLoS One. 2022 May 19;17(5):e0268496. doi: 10.1371/journal.pone.0268496 (PMC9119434; doi:10.1371/journal.pone.0268496)
Supplement: S2 Table — (PDF) [file pone.0268496.s002.pdf]

**S2 Table. Primer sequences for SNPs (*rs3844283* and *rs708035*)**			
**SNP *rs3844283***			
**Primers**	**Sequence**	**Melting Temperature**	**Product Size**
**FI (C allele)**	AGAGGATTTCATCCGGGTGGGGCTGC	76℃	127 bp
**RI (G allele)**	CAGCTGAAGATGTCCACTCGCTTTGTGAC	74℃	175 bp
**FO**	TGGACCAAAATCTCACCCCCAAACTTGC	74°C	247 bp
**RO**	CAAAGGAGCCGTGGGAAGGACAAATCAA	74°C	
**SNP *rs708035***			
**Primers**	**Sequence**	**Melting Temperature**	**Product Size**
**FI (A allele)**	TCCCACAGAAGGACTTACTCCTCAGTCAA	68℃	123 bp
**RI (T allele)**	AGAGCGAGGCGGTGCTGCTTGGATTA	74℃	169 bp
**FO**	TTCTTACGATGTCCGGTCGGTTTTCTGA	71℃	237 bp
**RO**	GCCCTTCTCCAGGTACTTCTGGCAGATC	71°C	
