# Supplementary figures and images for: Multi-locus SNP analyses of interleukin 1 receptor associated kinases 2 gene polymorphisms with the susceptibility to rheumatoid arthritis
Source: PLoS One. 2022 May 19;17(5):e0268496. doi: 10.1371/journal.pone.0268496 (PMC9119434; doi:10.1371/journal.pone.0268496)

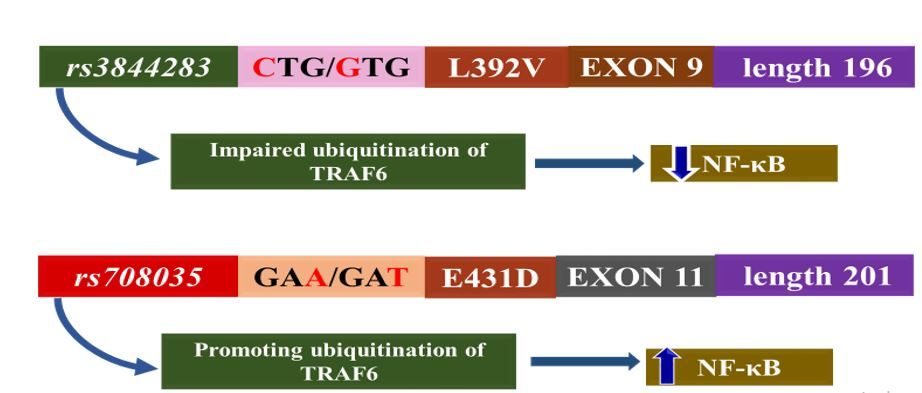

Supplement: S1 Appendix — (TIF) [file pone.0268496.s003.tif]
